# Design of Experiment (DoE) Approach for Developing Inhalable PLGA Microparticles Loaded with Clofazimine for Tuberculosis Treatment

**DOI:** 10.3390/ph17060754

**Published:** 2024-06-07

**Authors:** Druva Sarika Rongala, Suyash M. Patil, Nitesh K. Kunda

**Affiliations:** Department of Pharmaceutical Sciences, College of Pharmacy and Health Sciences, St. John’s University, Jamaica, NY 11439, USA; druvasarika.barji19@my.stjohns.edu (D.S.R.); suyash.patil19@my.stjohns.edu (S.M.P.)

**Keywords:** *Mycobacterium tuberculosis*, dry powder, inhalation, clofazimine, microparticle

## Abstract

Tuberculosis (TB) is an airborne bacterial infection caused by *Mycobacterium tuberculosis* (*M. tb*), resulting in approximately 1.3 million deaths in 2022 worldwide. Oral therapy with anti-TB drugs often fails to achieve therapeutic concentrations at the primary infection site (lungs). In this study, we developed a dry powder inhalable formulation (DPI) of clofazimine (CFZ) to provide localized drug delivery and minimize systemic adverse effects. Poly (lactic acid-co-glycolic acid) (PLGA) microparticles (MPs) containing CFZ were developed through a single emulsion solvent evaporation technique. Clofazimine microparticles (CFZ MPs) displayed entrapment efficiency and drug loading of 66.40 ± 2.22 %w/w and 33.06 ± 1.45 µg/mg, respectively. To facilitate pulmonary administration, MPs suspension was spray-dried to yield a dry powder formulation (CFZ SD MPs). Spray drying had no influence on particle size (~1 µm), zeta potential (−31.42 mV), and entrapment efficiency. Solid state analysis (PXRD and DSC) of CFZ SD MPs studies demonstrated encapsulation of the drug in the polymer. The drug release studies showed a sustained drug release. The optimized formulation exhibited excellent aerosolization properties, suggesting effective deposition in the deeper lung region. The in vitro antibacterial studies against H37Ra revealed improved (eight-fold) efficacy of spray-dried formulation in comparison to free drug. Hence, clofazimine dry powder formulation presents immense potential for the treatment of tuberculosis with localized pulmonary delivery and improved patient compliance.

## 1. Introduction

Tuberculosis (TB) is a chronic infectious bacterial disease that resulted in approximately 1.3 million deaths worldwide in 2022 alone [1]. TB is caused by *Mycobacterium tuberculosis* (*M. tb*), a pulmonary pathogen that enters the human body through the lungs via inhalation and causes pulmonary TB [1,2,3,4,5]. Clinically, TB is classified as latent TB infection (LTBI) and active TB infection [6]. Current therapy for active TB infection involves Directly Observed Treatment Short course (DOTS) with a combination of drugs that include rifampicin, isoniazid, ethambutol, and pyrazinamide with high intravenous doses of streptomycin for 6 to 9 months to cure the infection and prevent the spread [7]. However, with conventional therapy, a very minimal amount of drug reaches the lungs, requiring long-term treatment with high doses to maintain effective drug concentration in the lungs. In most cases, long-term treatment often leads to non-compliance that eventually causes the development of multi-drug resistant tuberculosis (MDR-TB). Because current treatment options are limited, reformulating an existing highly active antibacterial drug, such as clofazimine, in a new drug delivery system could be an intriguing approach to overcoming TB, specifically by targeting the infection site.

Clofazimine (CFZ) is a poorly water-soluble antibiotic and anti-inflammatory drug belonging to the riminophenazine class. CFZ is highly active against various mycobacteria, such as *Mycobacterium leprae*, *Mycobacterium avium complex* (*MAC*), and *M. tuberculosis*. CFZ is clinically approved for the treatment of leprosy and is also recommended as a second-line agent for MDR-TB treatment [8]. Though the mechanism of action of CFZ is not fully understood, a few studies suggest that its antibacterial effect is caused by the release of toxic lysophospholipid enzymes [9]. CFZ has also been used in combination with other anti-TB drugs, and there is an ongoing Phase-II clinical study of CFZ with a combination of delamanid, bedaquiline, and fluoroquinolones (norfloxacin and levofloxacin) to evaluate its therapeutic effectiveness in MDR-TB [10]. However, current clinical treatment of MDR-TB requires a 100 mg oral dose of CFZ [8]. Importantly, a minimum of 30 days of oral dosing is required to reach the steady-state concentration, thereby emphasizing the use of large loading doses and delay in anti-tuberculosis action [11]. The high oral doses often lead to severe adverse effects such as discoloration of the skin and conjunctiva, bioaccumulation in body fat tissue, cardiotoxicity, and severe GI distress like abdominal pain, nausea, diarrhea, and vomiting [12]. Additionally, the CFZ oral capsule is characterized by poor aqueous solubility and variable oral bioavailability (45 to 62%).

Pulmonary drug delivery could be an effective alternative as it allows for direct delivery of CFZ into the lungs and achieves effective lung concentrations [13,14]. In comparison to conventional oral drug delivery, it requires a lower dose, shorter treatment duration, and lower dosing frequency. Moreover, a large pulmonary surface area with thin alveolar epithelium offers the advantage of faster onset of drug action. Though several types of pulmonary delivery formulations and devices are available, dry powder inhalers have distinctive advantages of high stability, ease of handling, and portability over pressurized metered-dose inhalers, soft-mist inhalers, and nebulizers. Additionally, inhalable dry powder formulations containing microparticles can also take advantage of alveolar macrophage phagocytosis and target the mycobacteria residing in them. Previously, Brunaugh et al. explored the use of air jet milling to produce inhalable CFZ powder. However, their study lacked data pertaining to the stability and in vitro efficacy of CFZ formulation [8]. This is important as air jet milling often induces surface defects in the particles, leading to alterations in their crystalline structure, subsequently affecting stability and aerodynamic characteristics [15]. Here, we propose to develop a polymer-based inhalable CFZ powder via spray drying that is stable and efficacious against TB.

Polymer-based drug delivery systems have been explored extensively to improve solubility and increase the bioavailability of lipophilic drugs [16,17,18]. Poly (lactic acid-co-glycolic acid) (PLGA) is a biodegradable polymer that is safe for pulmonary delivery [19,20]. In addition, PLGA microparticles (MPs) offer improved drug loading and sustained drug release and allow for the passive targeting of alveolar macrophages [14,21]. Further, formulating PLGA MPs into dry powders will enhance stability and prevent drug leakage from MPs in suspension [22]. In this study, we have used a Design of Experiment (DoE) approach to systematically identify and optimize the spray drying process parameters for the successful production of CFZ-loaded PLGA MPs (CFZ SD MPs). Moreover, the resultant CFZ SD MPs were characterized for encapsulation efficiency, drug loading, and in vitro aerosolization performance. Additionally, the in vitro antibacterial activity of CFZ SD MPs was evaluated.

## 2. Results and Discussion

### 2.1. Characterization of CFZ MP

TB requires a prolonged oral treatment of 6 to 9 months, leading to dose-dependent toxicity and poor patient compliance. Our objective was to provide localized drug delivery to the lungs, minimize systemic side effects, and shorten the treatment duration. Toward that, we prepared clofazimine PLGA microparticles (CFZ MPs), followed by spray drying to formulate an inhalable dry powder of clofazimine.

Optimization of polymer MP size is important as it governs the uptake by alveolar macrophages [23,24]. A comparative study by Makino et al. observed that alveolar macrophages more efficiently took up microparticles with particle size around 1 µm than larger particles (6 or 10 µm) [25]. As TB resides in alveolar macrophages, the ideal particle size should be around 1 µm [26,27]. Homogenization speed and time are critical factors that significantly influence particle size [28,29]. Hence, we prepared MPs by varying homogenization parameters to achieve the target particle size of ~1 µm. For the first trial, MPs were prepared with a homogenization speed of 8000 rpm for 10 min (CFZMP001), which resulted in a mean particle size of 2.16 ± 0.15 µm with a zeta potential of −29.06 ± 0.51 mV (Table 1). For the second trial, the homogenization speed and time were increased to 10,000 rpm for 12 min, respectively (CFZMP002), and the particle size was reduced to 1.04 ± 0.08 µm with no significant change in zeta potential (Table 1). As the particle size was 1 µm, CFZMP002 was selected as an optimized clofazimine-loaded microparticle formulation (CFZ MPs) for spray drying optimization. The entrapment efficiency and drug loading of clofazimine in both formulations were ~66% and about ~33 µg/mg, respectively. Unlike particle size, the effect of homogenization speed and time was insignificant on the formulation’s entrapment efficiency and drug loading.

### 2.2. Spray Drying

The optimized clofazimine microparticles (CFZMP002) were spray-dried using L-leucine, an inert carrier, to prepare dry powders suitable for inhalation. L-leucine is an endogenous amino acid previously evaluated by us and many others for pulmonary administration as it provides improved flow, good aerosolization performance, and enhanced formulation dispersibility [30,31,32,33,34]. Recently, a DPI formulation for capreomycin containing L-leucine as a carrier was developed by Dharmadhikari et al. and was successfully administered for Phase-1 clinical study [35].

#### 2.2.1. Experimental Design

A Box–Behnken design was used to study the effects of various spray drying process parameters, such as inlet temperature, aspirator setting, and feed rate, on the powder yield and outlet temperature. The spray drying runs were performed according to the conditions mentioned in Table 2, as per Box–Behnken design.

The collected experimental data were fitted using sequential model analysis, and the best-fit model for yield was a linear model, and for outlet temperature, it was a quadratic model. The values of the R^2^, adjusted R^2^, SD, and the % CV (coefficient of variance) are given in following Table 3.

#### 2.2.2. Response Surface Mapping

##### Product Yield

**Figure 1:**
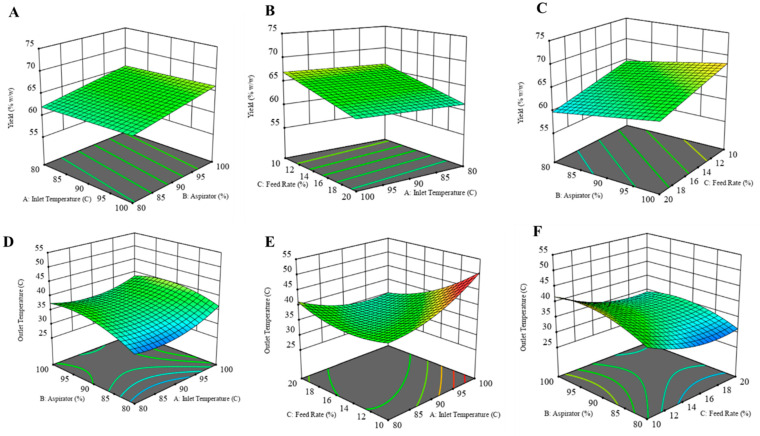
Three-dimensional (3D) response surface plots of (**A**) yield as a function of X1: inlet temperature and X2: aspirator, (**B**) yield as a function of X1: feed rate and X2: inlet temperature, (**C**) yield as a function of X1: Aspirator and X2: feed rate, (**D**) outlet temperature as a function of X1: aspirator and X2: inlet temperature, (**E**) yield as a function of X1: feed rate and X2: inlet temperature, (**F**) yield as a function of X1: Aspirator and X2: feed rate.

The yield of the formulation was calculated as the percent weight of solids obtained after spray drying to the initial amount of solids used to prepare the solution. While preparing the dry powder formulation, the goal was to achieve the highest yield while maintaining the outlet temperature below 42 °C. The 3D response surface plots for each dependent variable are illustrated in the figure below (Figure 1). Product yield was highest for CFZ08 (~71.01%), and the lowest yield was for CFZ06 (~56.71%). In Figure 1A, a linear relationship between product yield and inlet temperature was observed; on increasing the inlet temperature there was an increase in yield. Although the R^2^ value is lower, a fair amount of dry powder was recovered after spray drying for most of the formulations. Likewise, there was a significant increase in yield with an increase in aspirator setting (Figure 1A,C). The feed solution flowing through the nozzle of the spray dryer has an inverse relationship with the yield; a lower feed rate maximizes the yield of the spray-dried product (Figure 1B,C). Maury et al. also observed that the feed rate impacts the particle drying process; if the droplet proceeds at a higher feed rate, insufficient drying of particles causes particles to adhere, resulting in reduced powder yield [36]. A similar negative effect of higher feed rate on yield was observed by Focaroli et al. while optimizing the spray drying parameters for spray drying of L-leucine and trehalose formulations [37].

##### Outlet Temperature

The outlet temperature below 42 °C is a crucial parameter, as the temperature above that can cause a transition in PLGA structure [38]. The outlet temperature during the spray drying process is influenced by several factors, such as the inlet temperature, aspirator setting, and feed rate. Across 15 experimental runs, the outlet temperatures ranged between 28 and 46 °C. Graphical representations in the form of response surface plots revealed a U-shaped pattern, indicative of a quadratic model. Notably, the increase in inlet temperatures corresponded to increased outlet temperatures, particularly evident at lower feed rates (Figure 1D,E). Similarly, elevating the aspirator setting led to a rise in outlet temperature. Conversely, an increase in the feed rate resulted in a decrease in outlet temperature. This inverse relationship between feed rate and outlet temperature can be explained by the higher supply of liquid to the drying chamber at an increased feed rate, causing a greater generation of solvent vapor. Consequently, this surplus of vapor contributed to reducing the exhaust temperature at the outlet.

#### 2.2.3. Determination of Design Space

**Figure 2:**
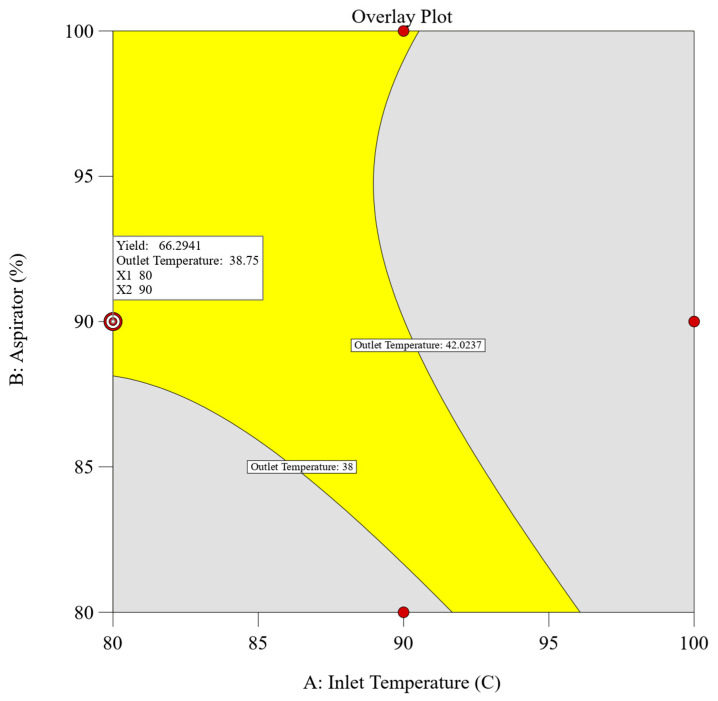
Overlay plot of experimental design for spray drying with defined target values.

A combined approach utilizing both numerical and graphical optimization methodologies was implemented to define the design space. The primary objective was to establish parameters that would maximize the dependent variables, specifically achieving the highest yield while ensuring that the outlet temperature remained below 42 °C (corresponds to the glass transition temperature of the polymer). Adhering to these predefined criteria, numerical optimization using design expert v13 software was employed, yielding multiple solutions characterized by desirability values. Among these solutions, the spray drying process parameters comprising an inlet temperature of 80 °C, an aspirator setting of 90%, and a feed rate of 10% achieved the highest desirability score of 0.875, meeting our target response. To further validate these findings, graphical optimization techniques were employed. An overlay plot was constructed to delineate the design space and confirm the results obtained through numerical optimization. Figure 2 illustrates the overlay plot, presenting the combination of independent parameters and the resultant values of the dependent parameters. Within the overlay plot, the yellow region signifies the designated design space, whereas the grey area shows the remaining experimental domain. Enclosed by contour lines, each boundary limit corresponds to the target set for the individual dependent parameters.

### 2.3. Optimized Dry Powder Formulation

The optimized dry powder formulation of clofazimine was prepared using a PLGA MPs-to-L-leucine ratio of 1:1.5 *w*/*w* with a total solid content of 2.5% *w*/*w*. The spray dryer with an inlet temperature of 80 °C, an aspirator of 90%, and a feed rate of 10% were employed. These parameters resulted in the outlet temperature of ~38 °C with a yield of 71 ± 3 %w/w, as reported in Table 4. The drug content in the optimized CFZ SD MP was 19.33 ± 3.24 µg/mg.

The resuspending index indicated the variability of particle size before and after spray drying, and the formulation demonstrated a resuspending index closer to 100%. Thus, this study emphasizes that the dispersion of spray-dried powder yields microparticles of comparable size to that before spray drying.

**Figure 3:**
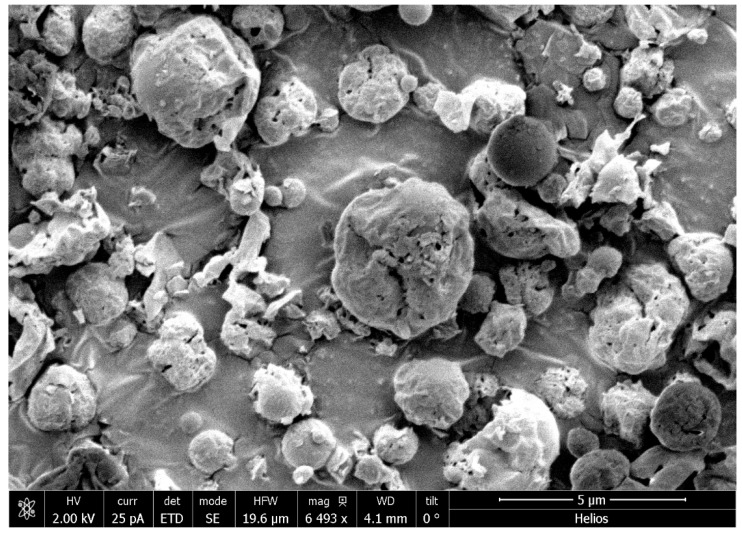
A Scanning Electron Microscope (SEM) image of optimized dry powder formulation of CFZ.

Scanning Electron Microscopy (SEM) was utilized to examine the morphology of CFZ SD MPs (Figure 3). The observed morphology of the spray-dried particles exhibited less spherical shapes with irregular and corrugated features. This irregularity in the particle structure is primarily attributed to the inclusion of L-leucine. This component contributes to the irregular shape, leading to enhanced dispersion characteristics of the powder within a dry powder inhaler (DPI). The irregular shape of the particles reduces inter-particulate contact points, subsequently lowering cohesive or adhesive forces compared to smoother particles [37].

### 2.4. Solid State Characterization (DSC, PXRD, TGA)

#### 2.4.1. Powder X-ray Diffraction (PXRD)

**Figure 4:**
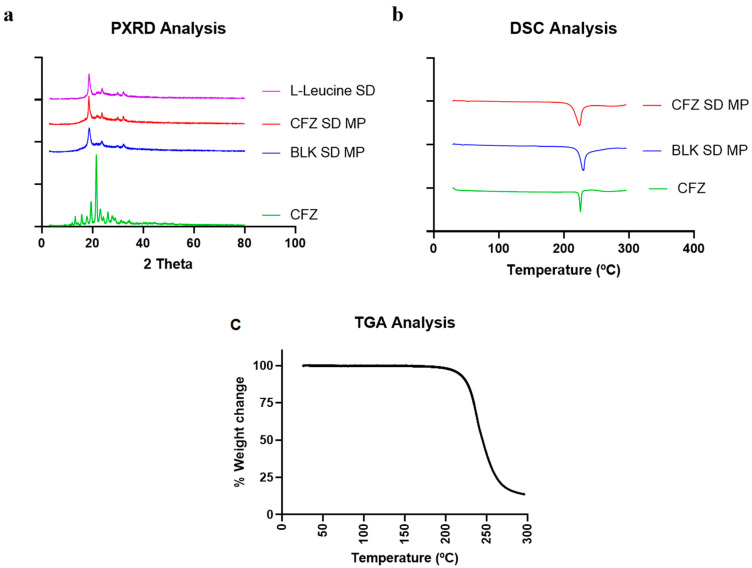
(**a**) Diffractogram of CFZ, CFZ SD MPs, and BLK SD MPs generated by Powder X-ray Diffraction (PXRD); (**b**) Thermograph of CFZ, CFZ SD MPs, and BLK SD MPs generated by Differential Scanning Calorimetry (DSC), and (**c**) TGA thermograph of CFZ SD MPs.

PXRD analyses were performed on spray-dried CFZ MPs to evaluate the crystalline nature of the formulation and confirm drug encapsulation. Figure 4a shows the PXRD diffractogram of the pure drug clofazimine (CFZ), spray-dried CFZ SD MPs, blank spray-dried microparticles (BLK SD MPs), and L-leucine. The results indicated that the pure drug CFZ had distinct sharp peaks at 2θ values of 20° and 21°, exhibiting the crystalline structure of the drug. Similar PXRD patterns and characteristic peaks of CFZ were reported by Brunaugh et al. [8]. The PXRD diffractograms of CFZ SD MPs and BLK SD MPs showed distinct peaks at 2θ values of 19°, 23°, and 30°, like spray-dried L-leucine alone. The semi-crystalline peaks observed in the spray-dried formulations are typical when using L-leucine as an excipient in spray drying and are extensively reported by others in the literature [39,40,41]. In addition, the drug characteristic peak at a 2θ value of 21° was absent in the diffractogram of CFZ SD MPs, confirming drug encapsulation.

#### 2.4.2. Differential Scanning Calorimetry and Thermogravimetric Analysis (DSC and TGA)

Figure 4b illustrates the DSC thermographs of CFZ, BLK SD MPs, and CFZ SD MPs. At 220 °C, the initial DSC thermograph of pure drug CFZ revealed a sharp endotherm [8], representing its melting point and confirming the crystalline nature of the drug. In this study, the CFZ SD MPs containing L-leucine as an inert carrier also showed endothermic transition at 218 °C. However, the DSC data of CFZ SD MPs seemed contrary to PXRD analysis (which suggests complete encapsulation of CFZ in the polymer). Therefore, to validate the absence of unentrapped drugs in microparticle formulation, we performed DSC of BLK SD MPs. The DSC thermograph of BLK SD MPs, which does not contain CFZ, also demonstrated an endothermic melting peak at 218 °C, suggesting that the endothermic peak might be due to other endothermic events. The DSC of BLK SD MPs indicated that the peak could be due to the presence of L-leucine. A similar phenomenon was observed by Tavares et al. in their work when developing PLGA dry powder microparticles for vaccine delivery to the lungs [42]. They observed that L-leucine underwent degradation at 218 °C, marked by a sharp melting endothermic peak on the DSC thermograph. To confirm this, we performed the TGA analysis of CFZ SD MPs. The TGA graph (Figure 4c) demonstrated that L-leucine underwent thermal degradation at 218 °C, marked by a steep decrease in the % weight of the sample. Therefore, we could assume that the endothermic peak at 218 °C in CFZ SD MPs was due to L-leucine. The above solid-state characterization studies thus reinforce the successful entrapment of CFZ into the microparticles.

### 2.5. In Vitro Release Study

**Figure 5:**
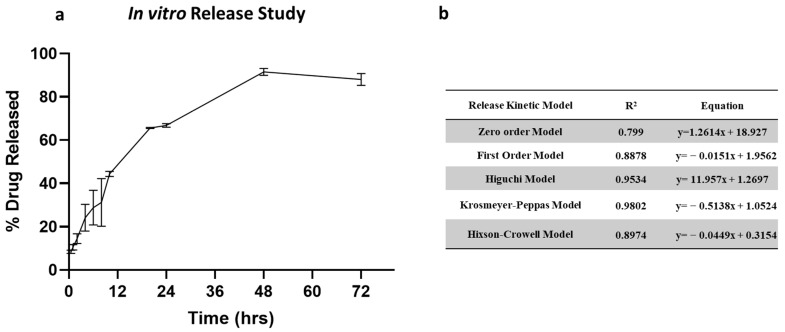
(**a**) In vitro release of CFZ from CFZ SD microparticles indicating burst release in 12 h and complete release within 72 h. (**b**) Summary of drug release kinetic models.

The in vitro release profile of clofazimine dry powder formulation is shown in Figure 5a. The release of CFZ from CFZ SD MP followed a biphasic release profile. The dry powder formulation showed an initial burst release of 44.34% in 12 h, followed by a slow and steady release for 72 h. The initial burst release occurs due to rapid CFZ release present in the outer region of the microparticles. In contrast, slow diffusion from the core of the microparticle occurs upon degradation of the PLGA polymeric matrix, resulting in sustained release. To understand the drug release kinetics, the release data were fitted into various release models, i.e., zero-order model, first-order model, Higuchi model, Krosmeyer–Peppas model, and Hixson–Croswell model. Figure 5b represents the equations and r^2^ values for each of the release models. According to the regression coefficient values, the best-fitted model is the Krosmeyer–Peppas model.

### 2.6. In Vitro Aerosolization Studies

Aerodynamic properties like mass median aerodynamic diameter (MMAD) and fine particle fraction (FPF) of the inhaled DPI formulations significantly impact the deposition rate and dosage. To deliver therapeutics into respiratory airways of the lungs, particles with MMAD of about 1 to 5 μm are essential. Particles deposited in the lower airways are taken up by alveolar macrophages where the TB bacteria reside [43].

**Figure 6:**
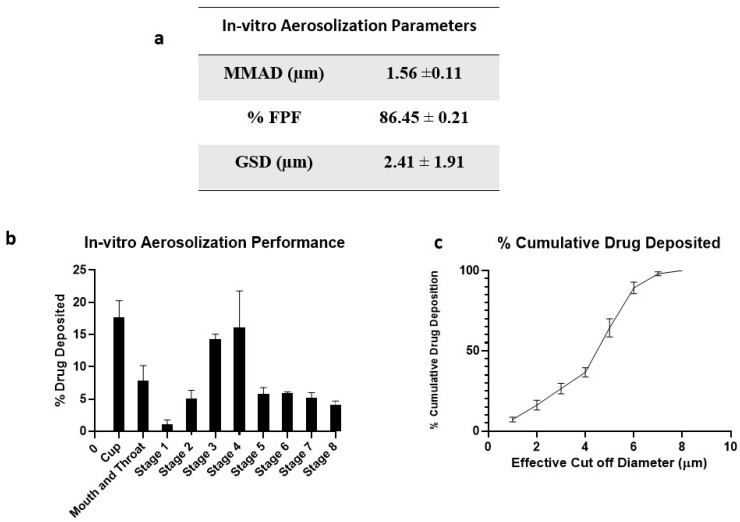
(**a**) In vitro aerosolization properties; (**b**) In vitro aerosol deposition profile of CFZ SD MPs represented as percent drug deposited on each stage of the Next Generation Impactor (NGI), and (**c**) % Cumulative drug deposition pattern. Data represented as Mean ± SD; *n* = 3.

Figure 6 represents the in vitro aerosolization properties, percentage of drug deposition on each stage of NGI, and percentage cumulative drug deposition pattern. The spray-dried formulation showed excellent aerosolization properties with an FPF of 86.45 ± 0.21%. The enhanced dispersibility of CFZ SD MPs could be attributed to the presence of L-leucine; it contains a hydrophobic alkyl side chain that alters the surface energy around the particle, thereby reducing the cohesion between particles [32]. A comparative study was conducted by Shetty et al. to evaluate the influence of various carriers like lactose, sucrose, trehalose, mannitol, and L-leucine on aerosolization and stability performance on ciprofloxacin formulations. They observed that L-leucine significantly increased the aerosol performance of ciprofloxacin with improved physical aerosol stability and FPF of about 70% compared to other carriers [44]. Similarly, Kunda et al. formulated nanocomposite microparticles with L-leucine as a carrier, and the formulations demonstrated excellent aerosolization properties [32]. Here, the mass median aerodynamic diameter (MMAD) value for CFZ SD MPs was 1.56 ± 0.11 µm, suggesting that the majority of the dose would deposit in the deeper lung regions (bronchial–alveolar). The geometric standard deviation (GSD) values of 2.41 ± 1.91 µm indicate that most of the emitted dose was polydisperse. A comparative study of monodisperse and polydisperse systems by Rosati et al. revealed improved lung deposition by polydisperse systems [45]. The spray-dried formulation of CFZ SD MPs displayed excellent aerodynamic properties, suggesting that a dry powder inhalation could be an ideal strategy for respiratory delivery of anti-mycobacterial therapeutics.

### 2.7. In Vitro Anti-Microbial Activity (MIC) Studies

**Figure 7:**
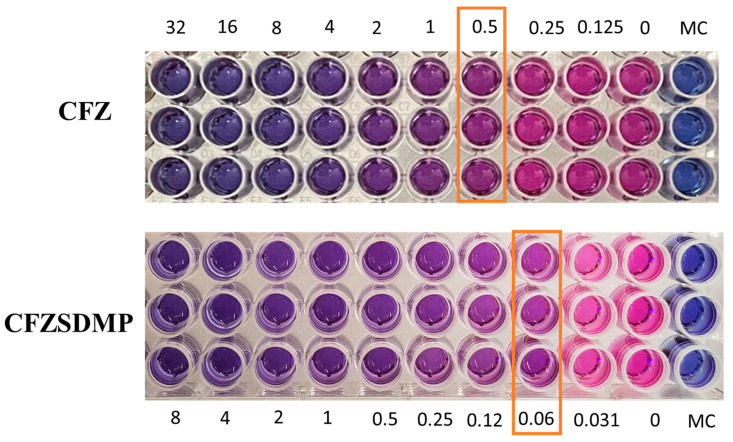
Resazurin-based microtiter plate assay (REMA) depicting color change from blue to pink as an indication of bacterial growth. The concentration where color change occurs is marked as minimum inhibitory concentration (MIC). Data represented as Mean ± SD, *n* = 3.

Resazurin colorimetric assay, also referred to as REMA, was utilized to evaluate the antibacterial activity (MIC Studies) of CFZ and CFZ SD MP on the H37Ra *Mycobacterium tuberculosis* strain. Resazurin undergoes oxidation and changes color from blue to pink during *M. tb.* Metabolism, indicating bacterial growth. However, the treatment groups with inhibited bacterial growth remain in blue. The MIC of the treatment group is the minimum concentration of the drug that allows for no more than 75% viability and prevents resazurin color change from blue to pink [46,47]. Figure 7 shows that the MIC of the free drug and CFZ SD MP on H37Ra was 0.25–0.5 µg/mL and 0.0625 µg/mL, respectively. The MIC study, thereby, revealed that the formulation (CFZ SD MPs) had improved antibacterial activity and was more effective in inhibiting bacterial growth than the free drug (Figure 7). In earlier reports by Lu et al. [48], the antibacterial activity of clofazimine on H37Rv was 0.12 to 0.24 µg/mL, like the MIC values we investigated [9,48]. Wengo et al. have spray-dried a combination of antibiotics (colistin and rifampicin) to prepare DPI and observed lower MIC for dry powder formulation than for free drug [49]. Therefore, the above findings potentiate the idea of effective DPI for treating respiratory infections.

### 2.8. Stability Studies

**Figure 8:**
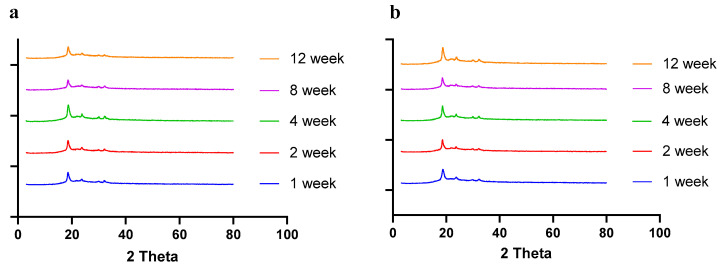
PXRD diffractograms of spray-dried CFZ SD microparticles during storage for 12 weeks at (**a**) 4 °C and (**b**) 25 °C.

**Figure 9:**
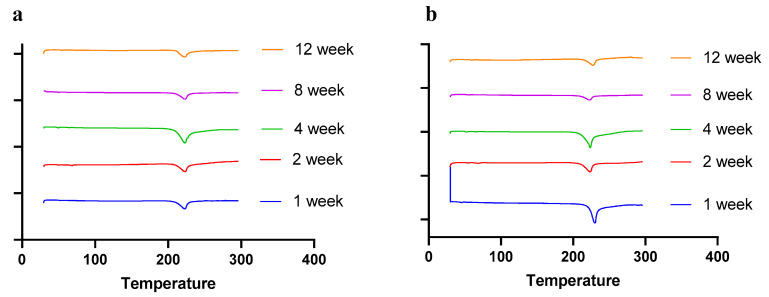
DSC thermographs of spray-dried CFZ SD microparticles during storage for 12 weeks at (**a**) 4 °C and (**b**) 25 °C.

Moisture, temperature, and storage time can alter the solid-state behavior of dry powder formulations, causing physical instability [50]. The presence of water (moisture) may increase the mobility of drug molecules, causing amorphous dry powder formulations to crystallize [51]. In a recent study by Shetty et al., it was observed that exposure to high humidity conditions led to the conversion of amorphous spray-dried drug particles of ciprofloxacin hydrochloride to re-crystallize, thereby altering the physical stability and aerosol performance [51]. Moreover, the solid state of the drug and excipients in the final formulation need to be consistent throughout the manufacturing process and storage, as solid-state transformation can alter the therapeutic efficacy of the formulation. Therefore, it is essential to evaluate the solid-state behavior during storage. To understand the effect of residual moisture, temperature, and storage time, we carried out stability studies and performed PXRD, DSC, and TGA studies. Herein, the spray-dried dry powder formulations were stored at different temperatures (4 °C and 25 °C) until 12 weeks. The PXRD and DSC studies showed no significant changes in the diffractogram and thermograph, respectively, concluding that the formulation maintained stability during storage over 12 weeks (Figure 8 (PXRD) and Figure 9 (DSC)).

Moreover, TGA analysis of dry powder formulations stored at 4 °C and 25 °C was also performed to determine the residual moisture content. The percent weight change from 60 °C to 100 °C marks the loss of moisture from the formulation. The TGA analysis demonstrated that the thermal mass loss was less than 3% after 8 and 12 weeks of storage, thereby confirming the absence of moisture and maintenance of the stability of the formulation.

To determine the effectiveness of the spray-dried formulation after storage at 4 and 25 °C, MIC studies were conducted at 4-week and 12-week time points (Table 5). The data revealed that the MIC of the formulation after storage was like the MIC before storage, thereby validating the stability of the formulation throughout storage.

## 3. Materials and Methods

### 3.1. Materials

Clofazimine was procured from Acros Organics (Morris Plains, NJ, USA). Poly (lactic-co-glycolic acid) (MW: 7000 to 15,000; 50:50) acid terminated was purchased from PolySciTech (West Lafayette, IN, USA). Polyvinyl alcohol (PVA) and L-leucine were purchased from Sigma-Aldrich (Saint Louis, MO, USA). Phosphate buffered saline (PBS) was obtained from Corning Inc. (Corning, NY, USA). Middlebrook broth 7H9 and Tween^®^ 80 were purchased from VWR International (Radnor, PA, USA). Dichloromethane (DCM, HPLC grade), Acetonitrile (ACN, HPLC grade), Water (HPLC grade), and Resazurin were procured from ThermoFisher Scientific (Waltham, MA, USA). Avirulent *Mycobacterium tuberculosis* strain (H37Ra) was procured from the American Type Culture Collection (ATCC 25177) (Manassas, VA, USA).

### 3.2. Preparation of CFZ-Loaded PLGA MPs

Clofazimine microparticles (CFZ MPs) were prepared using a previously published single emulsion solvent evaporation method with minor modifications [18,21]. Briefly, 100 mg of PLGA and 5 mg of CFZ were dissolved in 4 mL DCM to prepare the organic phase. The organic phase was then slowly added dropwise to 20 mL of 1% *w/v* PVA solution (aqueous phase) and was homogenized at 10,000 rpm for 12 min under ice to obtain an emulsion. The emulsion was subjected to stirring at 500 rpm for 3 h at room temperature to facilitate evaporation of the organic solvent and to harden the MPs. Further, the prepared microparticle suspension was centrifuged at 4 °C at 5150× *g* force for 10 min to obtain the final pellet. The pellet was washed twice with deionized water to remove the unentrapped drug. Finally, the washed pellet was redispersed in 4 mL of deionized water and stored at 4 °C until further use. Blank microparticles were prepared similarly without the drug.

#### 3.2.1. Characterization of CFZ PLGA MPs

##### Particle Size and Zeta-potential

The particle size, polydispersity index (PDI), and zeta potential were measured via dynamic light scattering using Malvern Zetasizer Nano ZS (Malvern Panalytical, Royston, UK). The microparticle suspension was diluted in a 1:75 ratio with deionized water and loaded into a cuvette for characterization. All the measurements were conducted at 25 °C in triplicate.

##### Entrapment Efficiency and Drug Loading

The concentration of clofazimine in MPs was determined using high-performance liquid chromatography (HPLC, Waters e2695 with 2489 UV/Vis Detector, Milford, MA USA) equipped with a C18 column (GL Sciences^®^ C18, 3 µm, 4.6 × 75 mm). The HPLC mobile phase consisted of 0.1% *v/v* orthophosphoric acid in HPLC grade water and acetonitrile (ACN) in a ratio of 25:75. The flow rate was maintained at 0.6 mL/min with an injection volume of 10 µL. Injected samples were detected at a UV wavelength of 284 nm. The chromatograms were obtained and analyzed using Empower 3.0 software (Waters, MA, USA). The drug concentrations were determined by establishing a standard calibration curve. The linearity range for the calibration curve was 0.1 ppm to 20 ppm. The equation of CFZ was found to be y = 125923x − 172.85, and the correlation coefficient was 0.999. The recovery studies indicated that the mean % recovery was 100.4%, with a precision value of no more than 2%. This indicates that the developed HPLC method is accurate and precise. The LOD and LOQ values for the method were 0.16 ppm and 0.48 ppm.

To determine the entrapment efficiency and drug loading, the drug was extracted from MPs, and the amount of CFZ encapsulated was determined using HPLC analysis. Briefly, 20 µL of MPs was added to 1 mL of ACN and 980 µL of deionized water, followed by centrifugation at 20,627× *g* force for 30 min at 4 °C. The supernatant was collected and analyzed using HPLC. The entrapment efficiency and drug loading were calculated using the following equations:

$$Entrapment\;Efficiency\;(\%\;w/w)\;=\;\frac{Amount\;of\;drug\;in\;CFZ\;MPs}{total\;amount\;of\;CFZ\;added}\times 100$$

$$Drug\;Loading(\frac{µg}{mg})=\frac{Amount\;of\;drug\;in\;CFZ\;MPs}{total\;amount\;of\;CFZ\;and\;polymer\;added}$$

### 3.3. Experimental Design

A Box–Behnken three factorial design (Design Expert v13) with three independent process variables was employed for optimization of the spray drying process. The process variables selected for the design were as follows: (1) inlet temperature; (2) feed rate; and (3) aspirator setting. Each process parameter was studied at three levels—low; medium; and high—by assigning −1, 0, and +1, as summarized in Table 6. The range of process variables was based on preliminary studies conducted individually by varying the inlet temperatures from 80 to 100 °C (the selected lower temperature ensured efficient drying of the particle, and the upper limit was selected to minimize the thermal degradation of formulation); aspirator setting between 80 to 100%, and feed rate of 10 to 20% were chosen to run the DoE.

### 3.4. Preparation of Spray-Dried CFZ MPs

The prepared microparticle suspension was spray-dried with an inert carrier, L-leucine, using Büchi mini-spray dryer B-290 (Büchi, Flawil, Switzerland) to obtain a dry powder formulation [30]. Briefly, the CFZ MPs were dispersed in L-leucine solution at a weight ratio of 1:1.5 (MP:L-Leu) to prepare the liquid feed for a total solid content of 2.5% *w*/*w*. Then, spray drying of the resultant liquid suspension was carried out at different inlet temperatures, feed rates, and aspirator settings, as listed in Table 6. The spray-dried powders were then collected from the cyclone and stored in a labeled glass vial at 4 °C until further use. Blank spray-dried microparticles were prepared similarly by spray-drying blank microparticle suspension.

#### 3.4.1. Spray Drying Product Yield

The dry powder obtained from spray drying was calculated based on the weight of powder collected expressed as a percentage of the weight of excipients and drugs introduced into the initial suspension of feed, providing the yield in percent by weight (%).

#### 3.4.2. Resuspendibility

About 1 mg of spray-dried powder was suspended in 1 mL of deionized water, and the particle size of NPs was determined as described in Section 3.2.1. The resuspendibility index was calculated as the % change in particle size before and after the spray drying.

### 3.5. Morphology

SEM images of spray-dried CFZ MPs were acquired using a Scanning Electron Microscope (FEI Helios Nanolab 660, ThermoFisher, MA, USA), as published by us before [52]. Briefly, the samples were sprinkled on the stub and sputter coated with gold. The particles were visualized using an electron beam at 2 kV with magnifications of 5000× and 12,000×.

### 3.6. Solid-State Characterization of Clofazimine Spray-Dried MPs

#### 3.6.1. Differential Scanning Calorimetry (DSC)

DSC studies were carried out using DSC 6000 (PerkinElmer, Inc., Branford, CT, USA) equipped with an intra-cooler accessory, as published by us before [53]. Briefly, 2 to 4 mg of spray-dried clofazimine microparticles (CFZ SD MPs), plain clofazimine (CFZ), and spray-dried blank PLGA microparticles (BLK SD MPs) were weighed and sealed in an aluminum pan. To obtain the thermal transition behavior and crystallinity, samples were heated from 30 °C to 300 °C at a constant heating rate of 10 °C/min. The DSC chamber was continuously purged with dry nitrogen at a flow rate of 50 mL/min throughout the analysis, and an empty aluminum pan was used as a reference.

#### 3.6.2. Powder X-ray Diffraction (PXRD)

The crystallinity of spray-dried formulations was evaluated by powder X-ray diffraction using a previously published method [54]. Dry powder samples (CFZ SD MPs, plain CFZ, and BLK SD MPs) were uniformly dispersed onto the sample holder of XRD-6000 (Shimadzu, Kyoto, Japan), and corresponding diffractograms were recorded using a continuous scan mode (scan speed of (2θ)/minute) at an angular range of 3–80°.

#### 3.6.3. Thermogravimetric Analysis (TGA)

The residual moisture content in the spray-dried powders was determined using a TGA Model Q50 (TA Instruments Inc., New Castle, DE, USA). Approximately 4 mg of spray-dried powder was weighed in a platinum sample holder and analyzed by heating the sample at a rate of 10 °C/min from 25 °C to 200 °C. The weight loss between 25 and 100 °C was represented as a loss of moisture in the dry powder formulation.

### 3.7. In Vitro Release Study

The in vitro drug release from dry powder microparticles was performed in phosphate-buffered saline (PBS, pH 7.4) containing 2% sodium dodecyl sulfate using a previously reported method [55]. About 10 mg of dry powder formulation was dispersed in 20 mL of release medium and subjected to continuous stirring at 100 rpm. The temperature of the release medium was maintained at 37 ± 0.5 °C. At pre-determined time points, 2 mL of release samples were collected and centrifuged at 20,627× *g* force for 30 min. Later, supernatants were collected and analyzed by HPLC. After, 2 mL of fresh release medium was used to suspend the MPs pellet and was added back into the release study to maintain sink conditions and facilitate further release of drug from MPs.

### 3.8. In Vitro Aerosolization Study

The in vitro deposition pattern of CFZ SD MPs was studied using a Next Generation Impactor (NGI) M170 (MSP Corporation, MN, USA). The NGI was equipped with a stainless-steel induction port, throat adaptor, and eight stages. Briefly, 10 mg of dry powder was loaded into a size 3 hypromellose capsule (Capsugel, Vcaps, Lonza, Morristown, NJ, USA) [56]. Afterward, the capsule was loaded onto an RS01^®^ dry powder inhaler (Plastiape, Italy), and the dry powder was aerosolized into NGI for 4 s at a flow rate of 60 L/min. After aerosolization, all stages (i.e., stages 1–8), including throat and induction port, were washed using a mixture of ACN and water (1:1) to dissolve and collect the drug. The amount of drug present on each plate, throat, induction port, and drug left in the capsule was subsequently analyzed using HPLC. The amount of drug deposited in each stage was used to calculate mass median aerodynamic diameter (MMAD) and fine particle fraction (FPF, %). MMAD was calculated using log-probability analysis, and FPF was calculated as the ratio of the amount of drug deposited in the NGI from stage 3–8 (d_ae_ < 4.46 µm) to the total emitted dose. The experiments were performed in triplicate, and data were presented as mean ± SD.

### 3.9. Stability Studies

The stability of dry powders was evaluated by storing the powders at 4 °C and 25 °C/~40% RH for 12 weeks. The samples were withdrawn at predetermined time intervals (1, 2, 4, 8, and 12 weeks) and evaluated using solid-state characterization studies (DSC and PXRD), as described in Section 3.6.1 and Section 3.6.2. In addition, the moisture content in the samples was also analyzed by TGA after 4 and 8 weeks, as described in Section 3.6.3.

### 3.10. Resazurin-Based Microtiter Plate Assay

The effectiveness of the CFZ SD MPs against *M. tuberculosis* H37Ra was measured using a resazurin-based microtiter plate assay (REMA). Briefly, the avirulent strain of *Mycobacteria tuberculosis* (H37Ra) was allowed to grow in 7H9 broth maintained at 37 °C by subjecting the culture medium to continuous stirring until the bacterial suspension reached an optical density (OD, 600 nm) of 1 (corresponding to 3.13 × 10^7^ colony forming units (CFU)/mL). The REMA plate assay was performed according to the previously reported literature [46,57,58]. About 100 µL of 7H9 broth was placed in each well of the 96-well plate, and serial two-fold dilutions of concentrations (64 µg/mL to 0.031 µg/mL) of CFZ and CFZ SD MPs were prepared directly in the plate. A bacterial control and media control (sterile control) were also included on the plate. The microplate was sealed with a gas-permeable polyethylene bag, followed by incubation at 37 °C for six days. After 6 days, 25 µL of resazurin: Tween^®^ 80 mixture (1:1) was added to the plate and re-incubated for an additional 24 h. The absorbance values were recorded using the plate reader, and bacterial viability was calculated by normalizing the absorbance values at 575 nm and 610 nm against the control. The minimum inhibitory concentration (MIC) was calculated as the lowest concentration that did not result in visible bacterial growth with less than 70% viability. The MIC for each sample (fresh MPs and MPs subjected to stability studies) was determined after three independent experiments, and the results were reported as a range.

## 4. Conclusions

In conclusion, clofazimine spray-dried microparticles were successfully formulated via spray drying. The spray-dried CFZ formulation demonstrated excellent aerosolization properties with a mass median aerodynamic diameter of 1.56 ± 0.11 µm and a high fine particle fraction of 86.45 ± 0.21%. Moreover, CFZ SD MPs demonstrated sustained drug release for 72 h with stability at 4 °C and 25 °C for 12 weeks. Further, the in vitro antibacterial studies showed improved potency of the CFZ spray-dried microparticles against H37Ra compared to free drugs. The above results demonstrate the immense inhalation potential of clofazimine dry powder formulation for treating tuberculosis.

## Figures and Tables

**Table 1:** Particle characterization of CFZ PLGA microparticles. All data presented as Mean ± SD; *n* = 3.

MP Sample	Particle Size (µm)	Zeta Potential (mV)	% Entrapment Efficiency (%w/w)	Drug Loading µg/mg)
CFZMP001 @8000 rpm 10 min	2.16 ± 0.15	−29.06 ± 0.51	66.03 ± 2.04	33.62 ± 1.05
CFZMP002 @10,000 rpm 12 min	1.04 ± 0.08	−31.42 ± 5.35	66.40 ± 2.22	33.06 ± 1.45

**Table 2:** Box–Behnken experimental design with observed responses.

S. No.	Independent Process Parameters			Dependent Product Parameters	
SD Sample ID	Inlet Temperature (°C)	Feed Rate (%)	Aspirator Setting (%)	Yield (% *w*/*w*)	Outlet Temperature (°C)
CFZ01	100	15	100	64.44	41
CFZ02	100	10	90	66.75	46
CFZ03	80	20	90	63.86	46
CFZ04	80	15	100	61.95	36
CFZ05	90	20	100	63.84	31
CFZ06	90	20	80	56.71	29
CFZ07	90	10	80	61.58	40
CFZ08	80	10	90	71.01	38
CFZ09	90	15	90	61.92	36
CFZ10	80	15	80	60.38	29
CFZ11	90	15	90	67.72	36
CFZ12	100	20	90	63.20	36
CFZ13	100	15	80	65.21	38
CFZ14	90	10	100	68.04	44
CFZ15	90	15	90	65.52	36

**Table 3:** Summary of regression analysis for the observed responses.

Responses	Model	R^2^	Adj R^2^	SD	%CV
Yield	Linear	0.4396	0.2868	2.97	4.62
Outlet Temperature	Quadratic	0.7659	0.3446	4.35	11.60

**Table 4:** Spray drying parameters for preparation of CFZ MPs dry powder and characterization. All data presented as Mean ± SD; *n* = 3.

SD Sample	Inlet Temperature. (°C)	Pump %	Aspirator %	% Yield (%w/w)	Outlet Temperature (°C)	Drug Content (µg/mg)	Resuspending Index (%)
CFZ08	80	10	90	71.01 ± 3.67	38	19.33 ± 3.24	99.7%

**Table 5:** MIC values of CFZ-free drug and spray-dried formulation (CFZ SD MPs) after storage up to 12 weeks at 4 and 25 °C.

MP Sample	MIC	MIC 4-Week 4 °C	MIC 12-Week 4 °C	MIC 12-Week 25 °C
CFZ-Free Drug	0.25 to 0.5 µg/mL			
CFZ SD MPs	0.0625 µg/mL	0.0625 µg/mL	0.0625 µg/mL	0.125 µg/mL

**Table 6:** Independent process variables and their coded values used in Box–Behnken design.

Independent Process Variables			
Levels	Low (−1)	Medium (0)	High (+1)
INLET TEMPERATURE (℃)	80	90	100
ASPIRATOR (%)	80	90	100
FEED RATE (%)	10	15	20

## Data Availability

Data are contained within this article.
